# Antibiotic-Resistant Bacteria in Green Turtle (*Chelonia mydas*) Rearing Seawater

**DOI:** 10.3390/ani11061841

**Published:** 2021-06-21

**Authors:** Thanaporn Chuen-Im, Korapan Sawetsuwannakun, Pimmnapar Neesanant, Nakarin Kitkumthorn

**Affiliations:** 1 Department of Microbiology, Faculty of Science, Silpakorn University, Nakhon Pathom 73000, Thailand; k.sawetsuwannakun@hotmail.com; 2 2/4 Suan Duang Pohn Village, Bang Khanun, Bang Kruai, Nonthaburi 11130, Thailand; pimmnapatr.n@gmail.com; 3 Department of Oral Biology, Faculty of Dentistry, Mahidol University, Bangkok 10400, Thailand; nakarinkit@gmail.com

**Keywords:** antibiotic resistant bacteria, bacterial infection, seawater, conservation, green turtle, *Chelonia mydas*

## Abstract

**Simple Summary:**

The Sea Turtle Conservation Center of Thailand (STCCT) has conducted an early intervention program for conservation and faced high mortality rates due to bacterial diseases. Our previous investigation of juvenile turtle carcasses and sea water in the turtle hold tanks implied an association between bacterial isolates in rearing water and infection in captive turtles. In this study, for a management plan of juvenile sea turtles with bacterial infection, we monitored antibiotic resistance of bacteria in seawater from juvenile green turtle holding tanks at STCCT in three periods: January 2015 to April 2016, January to April 2018, and January to April 2019. The results clearly indicated that numbers of resistant bacteria and antibiotics were increased. Assessment of resistance against ten antibiotics revealed high prevalence of antibiotic-resistant bacteria to the beta-lactam class (ampicillin, penicillin, and cefazolin), whereas low resistant isolate numbers were found to aminoglycosides. From the results of this study, we suggest that antibiotic-resistant bacterial assessment in sea turtle rearing seawater will provide important information for the treatment of bacteria-infected sea turtles in husbandry.

**Abstract:**

Antibiotic resistance of microorganisms is a serious health problem for both humans and animals. Infection of these bacteria may result in therapy failure, leading to high mortality rates. During an early intervention program process, the Sea Turtle Conservation Center of Thailand (STCCT) has faced high mortality rates due to bacterial infection. Previously, investigation of juvenile turtle carcasses found etiological agents in tissue lesions. Further determination of sea water in the turtle holding tanks revealed a prevalence of these causative agents in water samples, implying association of bacterial isolates in rearing water and infection in captive turtles. In this study, we examined the antibiotic resistance of bacteria in seawater from the turtle holding tank for a management plan of juvenile turtles with bacterial infection. The examination was carried out in three periods: 2015 to 2016, 2018, and 2019. The highest isolate numbers were resistant to beta-lactam, whilst low aminoglycoside resistance rates were observed. No gentamicin-resistant isolate was detected. Seventy-nine isolates (71.17%) were resistant to at least one antibiotic. Consideration of resistant bacterial and antibiotic numbers over three sampling periods indicated increased risk of antibiotic-resistant bacteria to sea turtle health. Essentially, this study emphasizes the importance of antibiotic-resistant bacterial assessment in rearing seawater for sea turtle husbandry.

## 1. Introduction

The Sea Turtle Conservation Center of Thailand (STCCT), operated by the Royal Thai Navy, has been conducting an early intervention program for the conservation of green turtles (*Chelonia mydas*) and hawksbill turtles (*Eretmochelys imbricata*). In the program, the sea turtle eggs were collected from the nests and incubated in safe places. After hatching, the juvenile turtles were raised, and released back into their natural habitat at the age of about 4–6 months. One major problem that occurred at STCCT while operating the conservation program was bacterial infection of the rearing turtles [1]. Identification of bacterial isolates from lesion tissues of juvenile turtle carcasses revealed that most etiologic agents belonged to the families *Vibrionaceae*, *Staphylococcaceae*, and *Enterobacteriaceae*, where the predominant genera were *Vibrio*, *Staphylococcus*, and *Citrobacter*. These bacteria were also commonly found in seawater from the juvenile green turtle rearing tanks [2]. Some of them are potential primary pathogens, and the others are opportunistic bacteria causing diseases in sea turtles. In addition to the observation at STCCT, high morbidity and mortality rates of both free-living and captive sea turtles due to bacterial infection have also been reported in other places, indicating that it is one of serious health problems of sea turtles [3,4,5,6,7,8,9,10].

Several publications have documented high numbers of antibiotic-resistant bacteria isolated from free-living sea turtles, including loggerhead turtles (*Caretta caretta*), black turtles (*Chelonia mydas agassizii*), olive ridley turtles (*Lepidochelys olivacea*), and green turtles (*Chelonia mydas*) [11,12,13,14,15,16,17]. Assessment of antibiotic resistance in bacteria isolated from hospitalized green turtles and water from animal holding tanks demonstrated that antibiotic-resistant bacterial acquisition in free-living sea turtles is partly from the contaminated environment, which leads to harder treatment [18]. More importantly, it has been shown to be associated with the death of animals [19,20]. As mentioned, bacterial diseases are one of the main causes of juvenile sea turtle death at STCCT [1]. Isolation and identification of bacterial isolates from the turtle carcasses and the rearing seawater has been performed to be used as fundamental information in terms of the prevention of potential diseases and rehabilitation of infected turtles. From the obtained results, it was possible that the prevalence of bacteria in rearing water was associated with the causative agents in infected turtles [1,2]. Recently, investigation of antibiotic-resistant bacteria in fish guts and pond water revealed that antibiotic resistance of bacterial isolates in fish guts were similar to those of bacterial isolates in the pond water [21]. The results from biochemical identification indicated that these bacteria were both non- and bacterial flora in the fish gut, implying a relation between antibiotic-resistant bacteria present in rearing water and in animals. The studies of Carini et al. and Agoba et al. provided evidence that antibiotic-resistant bacteria present in the habitat (e.g., rearing water in our case) can significantly affect raised animal health [18,21]. Therefore, the aim of this study was to investigate the current status and trend of antibiotic resistance of bacteria in juvenile turtle rearing seawater. The data obtained will provide important information for a management plan for the prevention and treatment of juvenile sea turtles with bacterial infections at STCCT (e.g., decision of antibiotic choice used for effective therapy).

## 2. Materials and Methods

### 2.1. Water Samples

**Figure 1:**
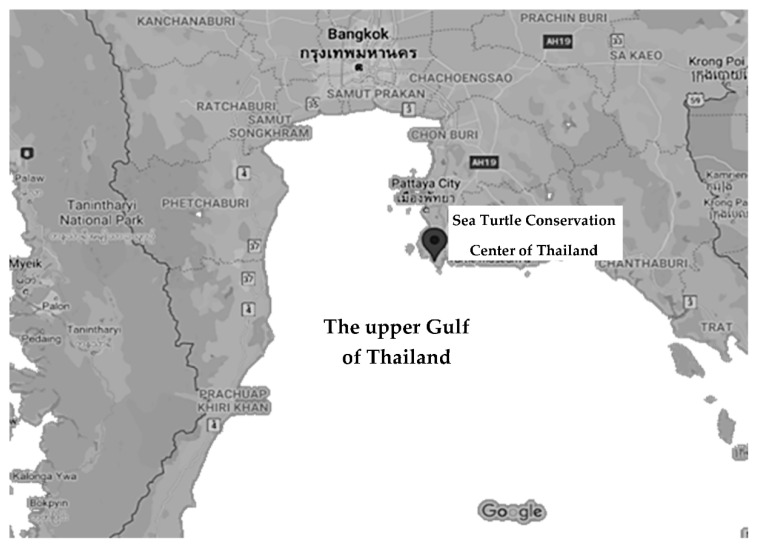
Location of the Sea Turtle Conservation Center of Thailand (STCCT). Source of map: Google Maps.

The Sea Turtle Conservation Center at Thailand (STCCT) is located at Sattahip District, Chonburi Province, Thailand (Figure 1). Water samples were collected from a juvenile green turtle rearing tank during the periods January 2015 to April 2016, January to April 2018, and January to April 2019. The tank was made of cement, with a size of 2 m depth · 2.5 m width · 2.5 m length, and lined with ceramic tiles. The water supply was pumped directly from the sea at a distance of 10–20 m offshore into the tank. The tank was used to rear juvenile green turtles (2–3 months of age) at a stocking density of 1.5–3.8 g/L with a static water system. The turtles were fed once daily with sea grapes (*Caulerpa lentillifera*) and twice daily with yellowstripe scad (*Selaroides leptolepis*). After feeding time lasted for 30 min, any uneaten food was discarded, and the water was drained entirely before rinsing the turtles in the tank with fresh seawater. The tank was cleaned, re-filled with fresh seawater, and then used to hold healthy juvenile turtles. Classification of the health of juvenile turtles was carried out by examining visible structures of the turtles (e.g., head, eyelids, skin, tegument, shoulder, flippers, tail, etc.) for signs of abscesses, wounds, and desquamation, as well as examining for slow movements and abnormal behavior [22]. After holding juvenile turtles for 3 hr, water samples were collected by using a 15 mL sterile tube. At a depth of 5 cm below the surface, the tube was carefully filled with water without any air remaining, before being placed on ice during transportation, and maintained at 4 °C until microbiological examination. The temperature of the water was measured at the time of sample collection using a glass thermometer, whereas pH and salinity were determined at the laboratory using a multi-parameter analyzer (Consort Medical, Hemel Hempstead, UK; Model C535).

### 2.2. Isolation and Identification of Bacteria in Juvenile Green Turtle Rearing Seawater

Isolation of bacteria was carried out by spreading water samples (0.1 mL each) on nutrient agar supplemented with 1% NaCl (NA + 1%NaCl), and then incubating at 25 °C for 25–48 h. Bacterial isolates were obtained by random selection of colonies, and then streaked on a NA + 1%NaCl plate to obtain pure culture. Each isolate was considered a separate organism, and identified using standard bacterial taxonomy procedures [23] as follows: Gram-staining, oxidase, catalase, gelatin hydrolysis, motility, carbohydrate fermentation, decarboxylase, urease, oxidation/fermentation, IMViC, triple sugar iron, citrate utilization, salt requirement and tolerance, and growth on various media including thiosulfate citrate bile salt sucrose agar, Baird Parker agar base, and eosin methylene blue agar. All media were purchased from HiMedia Laboratories (Mumbai, India).

### 2.3. Antibiotic Susceptibility Tests 

After identification, the isolates were tested for antibiotic activity using the disc diffusion method in accordance with the guidelines of the Clinical and Laboratory Standards Institute (CLSI) [24]. The bacteria were cultured in nutrient broth at 37 °C for 24 hr before being diluted into a concentration of 10^8^ CFU/mL. Then, lawn cultures were applied on Mueller Hinton Agar plates (HiMedia Laboratories, Mumbai, India), prior to being subjected to the antibiotic resistance test. Discs of ten antibiotics including aminoglycosides (streptomycin (10 μg), kanamycin (30 μg), gentamicin (10 μg), amikacin (10 μg), and tobramycin (10 μg)), beta-lactam (ampicillin (10 μg), penicillin (10 IU), cefazolin (30 μg)), and others (tetracyclin (30 μg) and chloramphenicol (30 μg)) (Oxoid, Basingstoke, Hants, UK) were placed on the surface of the agar plates. The plates were incubated for 24 h at 37 °C, and the inhibition zones were measured. *Escherichia coli* (ATCC 25922,) and *Staphylococcus aureus* (ATCC 25923,) were used as drug susceptibility controls. The bacterial strains were provided from the culture collection of the Department of Medical Sciences, Ministry of Public Health, Thailand.

## 3. Results

### 3.1. Temperature, pH, and Salinity of Seawater from Juvenile Green Turtle Holding Tank

Table 1 demonstrates abiotic parameters of water samples including temperature, pH, and salinity. The temperature of water samples during the periods 2015–2016, 2018, and 2019 ranged from 26.0 °C to 29.0 °C, 24.0 °C to 28.0 °C, and 26.0 °C to 28.0 °C, respectively. The range of water pH values observed in the periods 2015 to 2016, 2018, and 2019 was 7.2 to 7.9, 7.4 to 7.9, and 7.4 to 7.6, respectively. The salinity of water samples in the periods 2015 to 2016, 2018, and 2019 ranged from 25.2 to 35.0 ppt, 28.5 to 31.7 ppt, and 28.3–31.2 ppt, respectively. It was found that all parameters fluctuated throughout the year 2015 (January to December 2015).

### 3.2. Identification of Bacterial Isolates in Seawater from Juvenile Green Turtle Rearing Seawater

**Figure 2:**
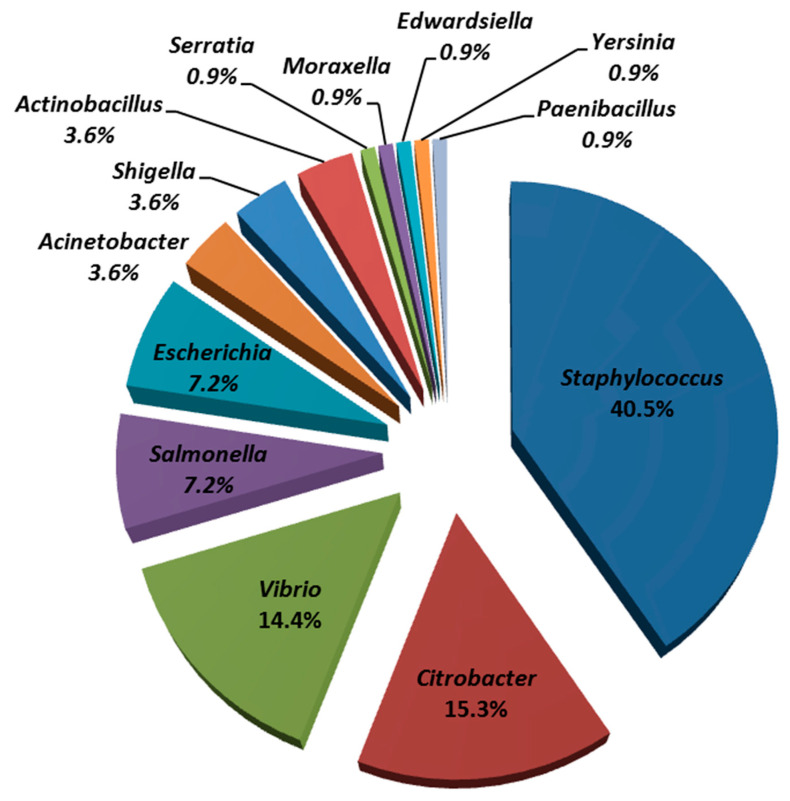
Percentages of bacterial genera (*n* = 111) isolated from seawater in juvenile green turtle holding tank at STCCT during the periods 2015 to 2016, 2018, and 2019.

A total of one hundred and eleven colonies were isolated from juvenile green turtle rearing water during the periods January 2015 to April 2016 (*n* = 85), January to April 2018 (*n* = 15), and January to April 2019 (*n* = 11). Using biochemical tests, it was found that the first, second, and third most common isolates belonged to *Staphylococcaceae* (45 isolates; 40.5%), *Enterobacteriaceae* (40 isolates; 36.0%), and *Vibrionaceae* (16 isolates; 14.4%), respectively. From the total of one hundred and eleven isolates, these isolates belonged to thirteen genera including *Staphylococcus*, *Citrobacter*, *Vibrio*, *Salmonella*, *Escherichia*, *Acinetobacter*, *Shigella*, *Actinobacillus*, *Serratia*, *Moraxella*, *Edwardsiella*, *Yersinia*, and *Paenibacillus* (Figure 2).

About 59.46% of the isolates (*n* = 66) were Gram-negative bacteria, where the first, second, and third most frequently identified bacteria were *Citrobacter* spp. (17 isolates), *Vibrio* spp. (16 isolates), *Escherichia coli,* and *Salmonella* spp. (8 isolates each), respectively. The remaining 45 isolates (40.54%) were Gram-positive bacteria and belonged to the genus *Staphylococcus* (Figure 2).

### 3.3. Resistance and Susceptibility of Bacterial Isolates to Ten Antibiotics

To determine antibiotic resistance and susceptibility of bacteria, the total one hundred and eleven isolates were tested against ten antibiotics: penicillin, cefazolin, ampicillin, chloramphenicol, tetracycline, amikacin, kanamycin, streptomycin, tobramycin, and gentamicin. From Table 2, the resistance of isolates to antibiotics can be ranked in descending order as follows: ampicillin (26.13%) > penicillin and cefazolin (21.62%) > chloramphenicol and tetracycline (12.61%) > amikacin (9.01%) > streptomycin (6.31%) > kanamycin and tobramycin (2.70%). For susceptibility, the percentages of isolates to antibiotics can be ranked as follows: gentamicin (100.00%) > tobramycin (95.50%) > kanamycin (89.19%) > amikacin (87.39%) > tetracycline (85.59%) > chloramphenicol (78.38%) > streptomycin (77.48%) > ampicillin (64.86%) > cefazolin (63.96%) > penicillin (48.65%). 

It was found that the first and second most *Staphylococcus* isolates were resistant to ampicillin (26.67%) and penicillin (22.22%), respectively (Table 2). All *Staphylococcus* isolates were susceptible to gentamicin, and the second and third most susceptible isolate numbers were to tobramycin and kanamycin (97.78% each) and streptomycin (95.56%), respectively. For *Enterobacteriaceae*, the first and second most resistant isolates were observed for cefazolin (30%) and ampicillin (25%), respectively. Similar to what was observed for *Staphylococcaceae*, all Enterobacteria isolates were susceptible to gentamycin. The second and third highest number of susceptible Enterobacteria numbers were found to tobramycin (97.50%), and amikacin and kanamycin (92.5% each), respectively. In the case of *Vibrio* spp., the highest isolate number was found for those resistant to penicillin and ampicillin (31.25% each). All *Vibrio* isolates were susceptible to gentamycin and chloramphenicol, and the second highest susceptible isolate number was observed for tobramycin (93.75%).

Table 3 demonstrates the patterns of antibiotic resistance observed in 111 isolates. Fifteen isolates (17.65%) were susceptible to all ten antibiotics. It should be noted that all of these isolates were isolated from water sample during the period 2015 to 2016. Intermediate resistance to one or two antibiotic(s) was detected in 17 isolates (16/85 and 1/11 isolates in years 2015 to 2016, and in 2019, respectively). Seventy-nine isolates (*n* = 111; 71.17%) were resistant to at one least antibiotic. Of these, there were 51 isolates (45.95%) resistant to only one antibiotic, which were penicillin, ampicillin, cefazolin, chloramphenicol, amikacin, tetracycline, and streptomycin. Resistance to tobramycin and kanamycin was seen in more than one antibiotic-resistant phenotype (patterns 10, 18, 21, 23, 24, and 25; Table 2). 

In this study, multi-antibiotic-resistant bacteria were defined as non-susceptible (intermediate or resistant) to at least one antibiotic in three or more antimicrobial categories [25]. Observation of multi-antibiotic resistance revealed 79 isolates (71.17%, *n* = 111). Of these, 54 isolates (63.53%, *n* = 85), 15 isolates (100.00%, *n* = 15), and 10 isolates (90.91%, *n* = 11) were detected in water samples during the periods 2015 to 2016, 2018, and 2019 (Table 3). There were two isolates, sampled in 2018, that displayed four antibiotic-resistant phenotypes (patterns 22–23). The highest antibiotic number found in multi-antibiotic-resistant bacteria was six antibiotics, observing in two isolates (*Paenibacillus* sp. and *Yersinia* sp.) from the years 2018 and 2019. 

## 4. Discussion

This study investigated antibiotic resistance of bacterial isolates from juvenile green turtle rearing water at the Sea Turtle Conservation Center of Thailand (STCCT) in the periods 2015 to 2016, 2018, and 2019. Juvenile green turtles at this center were cared for under the early intervention program for sea turtle conservation by collecting and incubating eggs in provided places, and raising the turtles in captivity until about four months of age, before releasing them back into the sea where the eggs were collected. Previously, we have demonstrated that bacterial infection in juvenile turtle carcasses at STCCT was associated with the prevalence of causative agents in rearing water [1,2]. The preliminary study on antibiotic-resistant bacteria in both coastal seawater, used as water supply at STCCT, and rearing seawater from the juvenile turtle holding tanks demonstrated similar resistance patterns to tested antibiotics (Table S1). To support rapid response and effective therapy for juvenile sea turtle treatment with bacterial infections, we determined the antibiotic resistance of bacteria in rearing water from the juvenile sea turtle holding tank. The results demonstrated that 71.17% of isolates were resistant to at least one of the ten tested antibiotics. Of these, 45.95% of bacterial isolates exhibited one antibiotic resistance (Table 2). It was also seen that the highest number of resistance to antibiotics observed in bacteria was increased from three antibiotics in the years 2015 to 2016 to six antibiotics in the years 2018 to 2019. A recent investigation of antibiotic resistance of Gram-negative bacteria from wild green turtles in Taiwan found that 89.36% of 47 isolates were resistant to at least one of 18 tested antibiotics [26]. Of these, 74.40% of bacteria were resistant to >2 antibiotics. Strikingly, the study of bacterial gut flora in green turtles revealed that all isolates of *Citrobacter* spp., the most common opportunistic bacterial isolates in green turtles, were resistant to at least one of twelve tested drugs [16]. This indicated that antibiotic resistance of bacteria is a threat to sea turtle health, and the severity tended to be increased.

In this study, antimicrobial resistance to five aminoglycosides including amikacin, kanamycin, streptomycin, tobramycin, and gentamicin were examined in bacteria isolated from juvenile green turtle rearing seawater. Compared with other classes, high susceptible rates of bacteria were detected in all aminoglycoside agents (Table 2). The results demonstrated that 100% of all tested isolates were susceptible to gentamicin, whereas the antibiotic susceptibility of the other four drugs were seen in an ordered ranking as follows: tobramycin (95.50%) > kanamycin (89.19%) > amikacin (87.39%) > streptomycin (77.48%). Gentamicin is one of aminoglycoside antibiotics that have been widely used for treatment not only in humans, but also terrestrial and aquatic animals [27,28,29]. In the past, the emergence of gentamicin-resistant bacteria was detected during the 1980s– 2000s, leading to the replacement of this drug by amikacin in the treatment of patients [30,31]. Among five aminoglycosides tested in this study, the most resistant isolates were observed for amikacin. In addition to this study, antibiotic resistance to gentamycin was also determined in isolates from free living sea turtles. Examination of *Vibrio* spp. from black turtles and olive ridley turtles found that >64% of *Vibrio parahaemolyticus* (*n* = 17) and *Vibrio cholera* (*n* = 6) were susceptible to gentamicin, whereas only 17.9% of *V. alginolyticus* (*n* = 39) were susceptible. Conversely, evaluation of antibiotic resistance patterns in juvenile loggerhead turtles in North Carolina revealed very high susceptibility rates to gentamicin [32]. Moreover, a study of antibiotic-resistant patterns of enterobacteria from seafood in the central areas of Thailand, where the seafood was mostly caught from the upper Gulf of Thailand, has reported non-detected bacterial isolates resistant to gentamicin [33]. This implies very low resistance rates of bacteria to gentamicin in the coastal seawater of the Sattahip area, a part of the upper Gulf of Thailand.

Recently, microbiological examination of juvenile green turtle holding tanks at STCCT found the predominant genera, *Vibrio*, *Staphylococcus*, and *Citrobacter,* in water samples [2]. These bacteria were mostly detected in tissue lesions of juvenile sea turtle carcasses [1]. Assessment of antibiotic resistance in this study demonstrated that bacterial isolates belonging to the families *Staphylococcaceae*, *Enterobacteriaceae*, and *Vibrionaceae* were highly susceptible to gentamicin, kanamycin, tobramycin, amikacin, and streptomycin (Table 2). Aminoglycosides are one of the most potent and broad-spectrum antibiotics that have been widely used in the treatment of various Gram-positive and Gram-negative bacterial infections, particularly members of *Enterobacteriaceae,* as well as *Staphylococcus aureus* [34]. The antibiotic agents specifically bind to ribosomal RNA subunits, leading to inhibition of protein synthesis [35]. However, gentamicin can induce nephrotoxicity, which may lead to acute renal failure [36]. Recently, plant-derived commercial dressing has been reported for use in loggerhead turtles with entanglement or severe head injury [37,38]. From the results in this study, we suggested that aminoglycosides together with plant-derived commercial dressing may be considered as a protocol to be used for treatment of bacterial infection in juvenile sea turtles at STCCT. 

The first and second highest number of antibiotic-resistant isolates from seawater in juvenile sea turtle holding tank at STCCT were observed for ampicillin, and penicillin and cefazolin, respectively. High prevalence of bacteria resistance to this antibiotic family has also been reported in both healthy and weak sea turtles by several publications [11,17,26,39,40,41,42]. The top three resistant antibiotics belonged to the beta-lactam group, where ampicillin was the most dominant drug, followed by penicillin and cefazolin. Incidence of high antibiotic-resistant rates of bacteria to beta-lactam agents was also observed in juvenile loggerhead turtles, green turtles, black turtles, and olive ridley turtles [12,14,32]. This may be because beta-lactam agents are commonly used to treat sea turtles with Gram-negative bacterial infections [26,43]. The high antibiotic resistance rates of microorganisms to these agents would affect the efficiency of treatment in infected turtles. Essentially, the results from this study have demonstrated that information on antibiotic resistance of bacteria in rearing water can contribute to management health plans for effective prevention and treatment of juvenile sea turtles with bacterial diseases (e.g., facility decolonization protocol, decision of antibiotic choice, emphasizing importance of antibiotic resistant bacteria monitoring as guidelines for sea turtle husbandry [19]). 

## 5. Conclusions

In conclusion, this study investigated the antibiotic resistance of bacteria in juvenile sea turtle rearing seawater in three periods: January 2015 to April 2016, January to April 2018, and January to April 2019. Although the sample numbers in 2018 and 2019 were much lower than those from 2015 to 2016 (*n* = 11, 15, and 85, respectively), investigation of multi-antibiotic-resistant bacteria clearly indicated that the situation of antibiotic resistance of bacteria in the juvenile green turtle holding tank at STCCT is likely to be increased in term of numbers of resistant antibiotics and bacteria (Table 2). This study revealed very low aminoglycoside resistance rates of bacteria in seawater from the holding tank, whereas high numbers of isolates were resistant to beta-lactam drugs, providing information for effective therapy of bacterial diseases at STCCT. Accordingly, antibiotic-resistant bacteria should be taken into account as a major health concern of sea turtles, and monitoring of these bacteria should be implemented in microbiological analysis of water quality for sea turtle husbandry.

## Figures and Tables

**Table 1:** Ranges of temperature, pH, and salinity values of seawater from juvenile green turtle holding tank at the Sea Turtle Conservation Center of Thailand (STCCT) during the periods 2015 to 2016, 2018, and 2019.

Year	Temperature (°C)	pH	Salinity (ppt)
January 2015 to April 2016	26.0–29.0	7.2–7.9	25.2–35.0
January to April 2018	24.0–28.0	7.4–7.9	28.5–31.7
January to April 2019	26.0–28.0	7.4–7.6	28.3–31.2

**Table 2:** Antibiotic susceptibility of one hundred and eleven isolates from turtle rearing seawater at STCCT during the periods 2015 to 2016, 2018, and 2019. The number in parentheses indicates percentages of isolates.

Antibiotic Phenotype *	PEN ^a^	AMP ^a^	CFZ ^a^	CMP ^a^	AMI ^a^	KA ^a^	S ^a^	TE ^a^	TOB ^a^	GEN ^a^
*Staphylococcaceae*										
S	20 (44.44)	31 (68.89)	37 (82.22)	31 (68.89)	41 (91.11)	44 (97.78)	43 (95.56)	40 (88.89)	44 (97.78)	45 (100.00)
I	15 (33.33)	1 (2.22)	1 (2.22)	6 (13.33)	0	0	0	0	0	0
R	10 (22.22)	12 (26.67)	7 (15.56)	8 (17.78)	4 (8.87)	1 (2.22)	2 (4.44)	5 (11.11)	1 (2.22)	0
Total (isolates)	45	45	45	45	45	45	45	45	45	45
*Enterobacteriaceae*										
S	25 (62.50)	27 (67.50)	21 (52.50)	36 (90.00)	37 (92.50)	37 (92.50)	25 (62.50)	35 (87.50)	39 (97.50)	40 (100.00)
I	13 (32.50)	3 (7.50)	7 (17.50)	0	0	2 (5.00)	13 (32.50)	1 (2.50)	0	0
R	2 (5.00)	10 (25.00)	12 (30.00)	4 (10.00)	3 (7.50)	1 (2.50)	2 (5.00)	4 (10.00)	1 (2.50)	0
Total (isolates)	40	40	40	40	40	40	40	40	40	40
*Vibrionaceae*										
S	8 (50.0)	7 (43.75)	11 (68.75)	16 (100.00)	12 (75.00)	12 (75.00)	12 (75.00)	13 (81.25)	15 (93.75)	16 (100.00)
I	3 (18.75)	4 (25.00)	5 (31.25)	0	2 (12.50)	4 (25.00)	3 (18.75)	0	1 (6.25)	0
R	5 (31.25)	5 (31.25)	0	0	2 (12.50)	0	1 (6.25)	3 (18.75)	0	0
Total (isolates)	16	16	16	16	16	16	16	16	16	16
*Moraxellaceae*										
S	0	4 (80.00)	0	1 (20.00)	4 (80.00)	4 (80.00)	3 (60.00)	4 (80.00)	4 (80.00)	5 (100.00)
I	2 (40.00)	1 (20.00)	1 (20.00)	4 (80.00)	1 (20.00)	1 (10.00)	0	0	0	0
R	3 (60.00)	0	4 (80.00)	0	0	0	2 (40.00)	1 (20.00)	1 (20.00)	0
Total (isolates)	5	5	5	5	5	5	5	5	5	5
*Pasteurellaceae*										
S	1 (25.00)	2 (50.00)	2 (50.00)	3 (75.00)	3 (75.00)	2 (50.00)	2 (50.00)	3 (75.00)	3 (75.00)	4 (100.00)
I	0	1 (25.00)	2 (50.00)	0	1 (25.00)	2 (50.00)	2 (50.00)	0	1 (25.00)	0
R	3 (75.00)	1 (25.00)	0	1 (25.00)	0	0	0	1 (25.00)	0	0
Total (isolates)	4	4	4	4	4	4	4	4	4	4
*Paenibacillaceae*										
S	0	0	0	0	0	0	1 (100.00)	0	1 (100.00)	1 (100.00)
I	0	0	0	0	0	0	0	1 (100.00)	0	0
R	1 (100.00)	1 (100.00)	1 (100.00)	1 (100.00)	1 (100.00)	1 (100.00)	0	0	0	0
Total (isolates)	1	1	1	1	1	1	1	1	1	1
Summary										
S	54 (48.65)	72 (64.86)	71 (63.96)	87 (78.38)	97 (87.39)	99 (89.19)	86 (77.48)	95 (85.59)	106 (95.50)	111 (100.00)
I	33 (29.73)	10 (9.01)	16 (14.41)	10 (9.01)	4 (3.60)	9 (8.11)	18 (16.22)	2 (1.80)	2 (1.80)	0
R	24 (21.62)	29 (26.13)	24 (21.62)	14 (12.61)	10 (9.01)	3 (2.70)	7 (6.31)	14 (12.61)	3 (2.70)	0
Total	111	111	111	111	111	111	111	111	111	111

^a^ PEN: penicillin; AMP: ampicillin; CFZ: cefazolin; CMP: chloramphenicol; GEN: gentamicin; AMI: amikacin; KA: kanamycin; S: streptomycin; TE: tetracycline; TOB: tobramycin. * S: susceptible; I: intermediate; R: resistant.

**Table 3:** Antibiotic-resistant, intermediate, and susceptible patterns of bacteria isolated from juvenile green turtle rearing water at STCCT during the periods January 2015 to April 2016, January to April 2018, and January to April 2019.

Pattern	Antibiotic Resistant Phenotype ^a^	Number of Isolates (%)			
		2015–2016 (*n* = 85)	2018 (*n* = 15)	2019 (*n* = 11)	Total (*n* = 111)
1	PEN	4 (4.71)	6 (40.00)	-	10 (9.01)
2	AMP	12 (14.12)	-	-	12 (10.81)
3	CFZ	8 (9.41)	-	4 (36.36)	12 (10.81)
4	CMP	2 (2.35)	5 (33.33)	-	7 (6.31)
5	AMI	5 (5.88)	-	-	5 (4.50)
6	TE	3 (3.53)	-	-	3 (2.70)
7	S	2 (2.35)	-	-	2 (1.80)
Number of isolates resistant to 1 antibiotic		36 (42.35)	11 (73.33)	4 (36.45)	51 (45.95)
8	PEN/AMP	3 (3.53)	2 (13.33)	-	5 (4.50)
9	AMP/TE	4 (4.71)	-	-	4 (3.60)
10	AMP/CFZ	2 (2.35)	-	-	2 (1.80)
11	PEN/CFZ	-	-	2 (18.18)	2 (1.80)
12	CMP/TE	1 (1.18)	1 (6.67)	-	2 (1.80)
13	AMP/AMI	1 (1.18)	-	-	1 (0.90)
14	CFZ/CMP	1 (1.18)	-	-	1 (0.90)
15	PEN/TOB	-	-	1 (9.09)	1 (0.90)
Number of isolates resistant to 2 antibiotics		12 (14.12)	3 (20.00)	3 (27.27)	18 (16.22)
16	PEN/AMP/CFZ	1 (1.18)	-	-	1 (0.90)
17	PEN/CFZ/AMI	1 (1.18)	-	-	1 (0.90)
18	PEN/AMP/KA	1 (1.18)	-	-	1 (0.90)
19	AMP/CMP/TE	1 (1.18)	-	-	1 (0.90)
20	CMP/AMI/TE	1 (1.18)	-	-	1 (0.90)
21	CFZ/TE/TOB	1 (1.18)	-	-	1 (0.90)
Number of isolates resistant to 3 antibiotics		6 (7.06)	-	-	6 (5.41)
22	CFZ/CMP/S/TE	-	-	1 (9.09)	1 (0.90)
23	PEN/S/TE/TOB	-	-	1 (9.09)	1 (0.90)
Number of isolates resistant to 4 antibiotics		-	-	2 (18.18)	2 (1.80)
24	PEN/AMP/CFZ/AMI/KA/S	-	1 (6.67)	-	1 (0.90)
25	PEN/AMP/CFZ/CMP/AMI/KA	-	-	1 (9.09)	1 (0.90)
Number of isolates resistant to 6 antibiotics		-	1 (6.67)	1 (9.09)	2 (1.80)
26	Intermediate resistance to 1 antibiotic	12 (14.12)	-	-	12 (10.81)
27	Intermediate resistance to 2 antibiotics	4 (4.71)	-	1 (9.09)	5 (4.50)
28	Susceptible to all ten antibiotics	15 (17.65)	-	-	15 (13.51)
-	Multi-antibiotic resistance ^b^	54 (63.53)	15 (100.00)	10 (90.91)	79 (71.17)

^a^ PEN: penicillin; AMP: ampicillin; CZL: cefazolin; CMP: chloramphenicol; GEN: gentamicin; AMI: amikacin; KAN: kanamycin; S: streptomycin; TE: tetracycline; TOB: tobramycin; -: not detected. ^b^ Multi-antibiotic resistance was defined as acquired non-susceptibility to at least one agent in at least three antimicrobial categories [25].

## Supplementary Materials

The following are available online at https://www.mdpi.com/article/10.3390/ani11061841/s1, Table S1 Total plate counts (log CFU/mL) and antibiotic resistant bacteria detected in coastal seawater and rearing seawater from the turtle holding tanks.

## References

[1] Chuen-Im T., Areekijseree M., Chongthammakun S., Graham S.V. (2010). Aerobic bacterial infections in captive juvenile green turtles (*Chelonia mydas*) and hawksbill turtles (*Eretmochelys imbricata*) from Thailand. Chelonian. Conserv. Biol..

[2] Chuen-Im T., Suriyant D., Sawetsuwanakun K., Kitkumthorn N. (2019). Occurrence of Vibrionaceae, Staphylococcaceae, and Enterobacteriaceae in green turtle *Chelonia mydas* rearing seawater. J. Aquat. Anim. Health.

[3] Wiles M., Rand T.G. (1987). Integumental ulcerative disease in a loggerhead turtle *Caretta caretta* at the Bermuda Aquarium: Microbiology and histopathology. Dis. Aquat. Org..

[4] Glazebrook J.S., Campbell R.S.F. (1990). A survey of the diseases of marine turtles in northern Australia. I. Farmed turtles. Dis. Aquat. Org..

[5] Glazebrook J.S., Campbell R.S.F. (1990). A survey of the diseases of marine turtles in northern Australia. II. Oceanarium-reared and wild turtles. Dis. Aquat. Org..

[6] Raidal S.R., Ohara M., Hobbs R.P., Prince R.I. (1998). Gram-negative bacterial infections and cardiovascular parasitism in green sea turtles (*Chelonia mydas*). Aust. Vet. J..

[7] Torrent A., Déniz S., Ruiz A., Calabuig P., Sicilia J., Orós J. (2002). Esophageal diverticulum associated with *Aerococus viridans* infection in a loggerhead sea turtle (*Caretta caretta*). J. Wildl. Dis..

[8] Greer L.L., Strandberg J.D., Whitaker B.R. (2003). *Mycobacterium chelonae* Osteroarthritis in a Kemp’s Ridley sea turtle (*Lepidochelys kempii*). J. Wildl. Dis..

[9] Orós J., Calabuig P., Déniz S. (2004). Digestive pathology of sea turtles stranded in the Canary Islands between 1993 and 2001. Vet. Rec..

[10] Orós J., Torrent A., Calabuig P., Déniz S. (2005). Diseases and causes of mortality among sea turtles stranded in the Canary Islands, Spain (1998-2001). Dis. Aquat. Org..

[11] Foti M., Giacopello C., Bottari T., Fisichella V., Rinaldo D., Mammina C. (2009). Antibiotic resistance of Gram negatives isolates from loggerhead sea turtles (*Caretta caretta*) in the central Mediteranean sea. Mar. Pollut. Bull..

[12] Al-Bahry S.N., Mahmoud I.Y., Al-Zadjali M., Elshafie A., Al-Harthy A., Al-Alwi W. (2011). Antibiotic resistant bacteria as bio-indicator of polluted effluent in the green turtles, *Chelonia mydas* in Oman. Mar. Environ. Res..

[13] Al-Bahry S.N., Al-Zadjali M.A., Mahmoud I.Y., Elshafie A.E. (2012). Biomonitoring marine habitats in reference to antibiotic resistance bacteria and ampicillin resistance determinants from oviductal fluid of the nesting green sea turtle. Chelonia mydas. Chemosphere.

[14] Zavala-Norzagaray A.A., Aguirre A.A., Velazquez-Roman J., Flores-Villaseñor H., León-Sicairos N., Ley-Quiñonez C.P., Hernández-Diaz L.D.J., Canizalez-Roman A. (2015). Isolation, characterization, and antibiotic resistance of *Vibrio* spp. in sea turtles from Northwestern Mexico. Front. Microbial..

[15] Pace A., Dipineto L., Fioretti A., Hochscheid S. (2019). Loggerhead sea turtles as sentinels in the western Mediterranean: Antibiotic resistance and environment-related modifications of Gram-negative bacteria. Mar. Pollut. Bull..

[16] Ahasan M.S. (2017). Gut Bacterial Communities in Health and Compromised Green Turtles (*Chelonia mydas*) and an Alternative Treatment for Gastrointestinal Disorders. Ph.D. Thesis.

[17] Alduina R., Gambino D., Presentato A., Gentile A., Sucato A., Savoca D., Filippello S., Visconti G., Caracappa G., Vicari D. (2020). Is *Caretta caretta* a carrier of antibiotic resistance in the Mediterranean Sea?. Antibiotics.

[18] Carini A.D.P., Ariel E., Picard J., Elliott L. (2017). Antibiotic resistant bacterial isolates from captive green turtles and *in vitro* sensitivity to bacteriophages. Int. J. Microbiol..

[19] Gili C., Biancani B., Gulland F., Mazzariol S. (2017). Meticillin-resistant *Staphylococcus aureus* (MRSA) associated dolphin mortality and the subsequent facility decolonisation protocol. Vet. Rec. Case Rep..

[20] Mazzariol S., Corrò M., Tonon E., Biancani B., Centelleghe C., Gili C. (2018). Death associated to methicillin resistant *Staphylococcus aureus* ST8 infection in two dolphins maintained under human care, Italy. Front. Immunol..

[21] Agoba E.E., Adu F., Agyare C., Boamah V.E., Boakye Y.D. (2017). Antibiotic resistance patterns of bacterial isolates from hatcheries and selected fish farms in the Ashanti region of Ghana. J. Microbial. Antimicrob..

[22] Reséndiz E., Lara-Uc M.M., Abubakar M. (2018). Health assessments in free-ranging sea turtles: Perspective of animal welfare in wildlife. Animal Welfare.

[23] Buchanan R.E., Gibbons N.E. (1974). Bergey’s Manual of Determinative Bacteriology.

[24] Clinical and Laboratory Standard Institute (CLSI) (2015). Performance Standards for Antimicrobial Disk Susceptibility Tests.

[25] Magiorakos A.P., Srinivasan A., Carey R.B., Carmeli Y., Falagas M.E., Giske C.G., Harbarth S., Hindler J.F., Kahlmeter G., Olsson-Liljequist B. (2012). Multidrug-resistant, extensively drug-resistant and pandrug-resistant bacteria: An international expert proposal for interim standard definitions for acquired resistance. Clin. Microbiol. Infect..

[26] Tsai M.A., Chang C.C., Li T.H. Multiple-antibiotic resistance of *Enterococcus faecalis* in an endangered olive ridley sea turtle (*Lepidochelys olivacea*): A case report. Indian J. Anim. Res..

[27] Kushner B., Allen P.D., Crane B.T. (2016). Frequency and demographics of gentamicin use. Otol. Neurotol..

[28] Lappin M.R., Blondeau J., Boothe D., Breitschwerdt E.B., Guardabassi L., Lloyd D.H., Papich M.G., Rankin S.C., Sykes J.E., Turnidge J. (2017). Antimicrobial use guidelines for treatment of respiratory tract disease in dogs and cats: Antimicrobial guidelines working group of the International Society for Companion Animal Infectious Disease. J. Vet. Intern. Med..

[29] Bojarski B., Jakubiak M., Bień M., Batoryna M., Formicki G., Socha M., Drąg-Kogzak E., Tombarkiewicz B. (2019). Assessment of gentamicin effect on oxidoreductive balance and microstructure of trunk kidney in Prussian carp (*Carassius gibelio*). Ann. Wars. Univ. Life Sci. SGGW Anim. Sci..

[30] Mulhern B., Griffin E. (1981). An epidemic of gentamicin/cloxacillin resistant staphylococcal infection in a neonatal unit. Ir. Med. J..

[31] Wielunsky E., Drucker M., Cohen T., Reisner S.H. (1983). Replacement of gentamicin by amikacin as a means of decreasing gentamicin resistance of gram-negative rods in a neonatal intensive care unit. Isr. J. Med. Sci..

[32] Harms C.A., Mihnovets A.N., Braun-McNeill J., Kelly T.R., Avens L., Goodman A., Goshe L., Godfrey M.H., Hohn A.A. Cloacal bacterial isolates and antimicrobial resistance patterns in juvenile loggerhead turtles in North Carolina, USA. Proceedings of the 26th Annual Symposium on Sea Turtle Biology and Conservation, International Sea Turtle Society.

[33] Pongsilp N., Nimnoi P. (2018). Diversity and antibiotic resistance patterns of enterobacteria isolated from seafood in Thailand. CYTA-J. Food.

[34] Krause K.M., Serio A.W., Kane T.R., Connolly L.E. (2016). Aminoglycosides: An overview. Cold Spring Harb. Perspect. Med..

[35] Kotra L.P., Haddad J., Mobasher S. (2000). Aminoglycosides: Perspectives on mechanisms of action and resistance and strategies to counter resistance. Antimicrob. Agents Chemother..

[36] Mingeot-Leclercq M.P., Tulkens P.M. (1999). Aminoglycosides: Nephrotoxicity. Antimicrob. Agents Chemother..

[37] Franchini D., Valastro C., Ciccarelli S., Ricciardi M., Lenoci D., Corrente M., Di Bello A. (2019). Assessment of residual vascularization of the limb as a prognostic factor to avoid sea turtle flipper amputation. J. Wildl. Dis..

[38] Franchini D., Cavaliere L., Valastro C., Carnevali F., Van der Esch A., Lai O., Di Bello O. (2016). Management of severe head injury with brain exposure in three loggerhead sea turtles Caretta caretta. Dis Aquat. Organ..

[39] Pinera-Pasquino L. (2006). Patterns of antibiotic resistance in bacteria isolated from marine turtles. Master’s Thesis.

[40] Blasi M.F., Migliore L., Mattei D., Rotini A., Thaller M.C., Alduina R. (2020). Antibiotic resistance of Gram-negative bacteria from wild captured loggerhead sea turtles. Antibiotics.

[41] Tsai M.A., Chang C.C., Li T.H. (2021). Antimicrobial-resistance profiles of Gram-negative bacteria isolated from green turtles (*Chelonia mydas*) in Taiwan. Environ. Pollut..

[42] Trotta A., Cirilli M., Marinaro M., Bosak S., Diakoudi G., Ciccarelli S., Paci S., Buonavoglia D., Corrente M. (2021). Detection of multi-drug resistance and AmpC β-lactamase/extended-spectrum β-lactamase genes in bacterial isolates of loggerhead sea turtles (*Caretta caretta*) from the Mediterranean Sea. Mar. Pollut. Bull..

[43] Innis C.J., Braverman H., Cavin J.M., Ceresia M.L., Baden L.R., Kuhn D.M., Frasca S., McGowan J.P., Hirokawa K., Weber E.S. (2014). Diagnosis and management of Enterococcus spp. infections during rehabilitation of cold stunned Kemp’s ridley turtles (*Lepidochelys kempii*): 50 cases (2006–2012). J. Am. Vet. Med. Assoc..

