# Supplementary material for: Antibiotic-Resistant Bacteria in Green Turtle (Chelonia mydas) Rearing Seawater
Source: Animals (Basel). 2021 Jun 21;11(6):1841. doi: 10.3390/ani11061841 (PMC8235308; doi:10.3390/ani11061841)
Supplement: Supplementary file 1 [file animals-11-01841-s001.zip › animals-1231259-supplementary.pdf]

animals

Article

# Antibiotic-Resistant Bacteria in Green Turtle (*Chelonia mydas*) Rearing Seawater

**Thanaporn Chuen-Im [1,*], Koraphan Sawetsuwannakun [1], Pimmnapar Neesanant [2] and Nakarin Kitkumthorn [3]**

**Table S1.** Total plate counts (log CFU/mL) and antibiotic resistant bacteria detected in coastal seawater and rearing seawater from the turtle holding tanks *.

| Total plate count ** (log CFU/mL) | Antimicrobial susceptibility | Gentamicin | Amikacin | Tobramycin | Tetracycline | Chloramphenicol | Ampicillin | Penicillin | Amoxicillin |
|---|---|---|---|---|---|---|---|---|---|
| Antibiotic resistant isolates from coastal seawater using as water supply (isolates) | | | | | | | | | |
| 2.85 | Resistant | 0 | 0 | 0 | 2 | 1 | 12 | 10 | 9 |
| | Intermediate | 0 | 1 | 1 | 0 | 0 | 0 | 2 | 0 |
| | Susceptible | 16 | 15 | 15 | 14 | 15 | 4 | 4 | 7 |
| | Total | 16 | 16 | 16 | 16 | 16 | 16 | 16 | 16 |
| Antibiotic resistant isolates from seawater that kept juvenile green turtles in the holding tank (isolates) | | | | | | | | | |
| 3.41 | Resistant | 0 | 0 | 2 | 6 | 2 | 28 | 25 | 25 |
| | Intermediate | 0 | 5 | 3 | 1 | 5 | 1 | 0 | 2 |
| | Susceptible | 33 | 28 | 28 | 26 | 26 | 4 | 8 | 6 |
| | Total | 33 | 33 | 33 | 33 | 33 | 33 | 33 | 33 |

* The preliminary study conducted during the end of 2014. ** NA + 3%NaCl.
